# Supplementary material for: Glucocorticoids intrinsically redirect naïve CD4+ T cells to the bone marrow for preservation in malnourished mice
Source: Biol Open. 2026 May 12;15(5):bio062485. doi: 10.1242/bio.062485 (PMC13225209; doi:10.1242/bio.062485)
Supplement: Supplementary information [file biolopen-15-062485-s1.pdf]

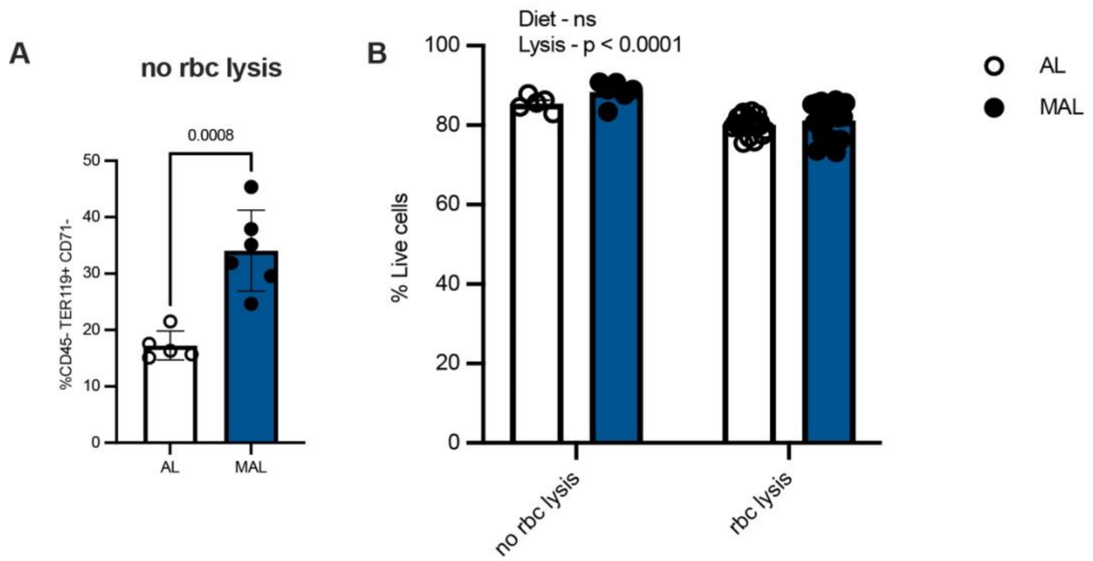

Fig. S1. Red blood cell lysis procedure in bone marrow sample preparation does not result in differential recovery of live cells from malnourished and control mice. Bone marrow was isolated from malnourished (MAL) and *ad libitum*-fed (AL) mice. Red blood cells were lysed in some samples (AL: *n*=22; MAL: *n*=21), while a smaller proportion were left untreated (AL: *n*=5; MAL: *n*=6). Flow cytometry was used to determine the percentage of late-stage erythroblasts (CD45-TER119+CD71-) in non-lysed bone marrow samples collected from malnourished and control mice (A). The difference between the percentage of CD45-TER119 +CD71- cells in AL and MAL samples was determined by a two-tailed unpaired *t*-test and the *P* value is indicated above the graph. Additionally, the percentage of live cells in both groups was determined by flow cytometry (B). *P* values were obtained by two-way ANOVAs and are displayed on graphs, with "Lysis" indicating the effect of red blood cell lysis and "Diet" indicating the effect of diet. n.s. - not significant. Graphs display means ± s.e.m.

Biology Open • Supplementary information

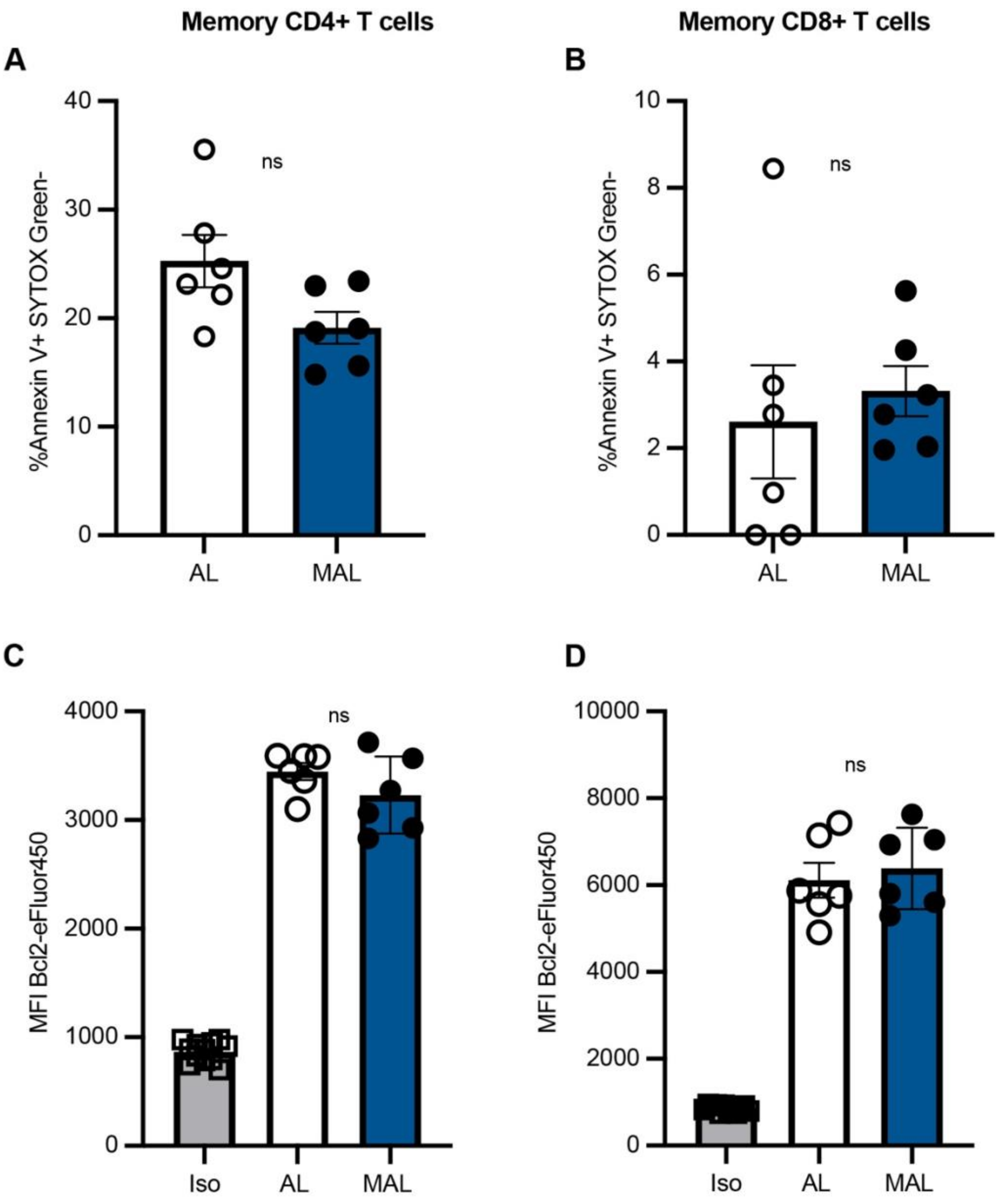

Fig. S2. Malnourished memory T cells undergo apoptosis in the bone marrow similarly to control cells. Flow cytometry was used to determine the percentage of Annexin V+ SYTOX Green- memory (CD44^{hi}) CD4+ (A) and CD8+ (B) T cells and the mean fluorescence intensity of Bcl2-eFluor450 of intracellularly stained memory (CD44^{hi}) CD4+ (C) and CD8+ (D) T cells isolated from the bone marrow of malnourished (MAL) and *ad libitum*-fed (AL) mice. Iso - isotype control. *n*=6/group. n.s. - not significant. *P* values, obtained by two-tailed unpaired *t*-tests, are indicated above each graph. Graphs display means ± s.e.m.

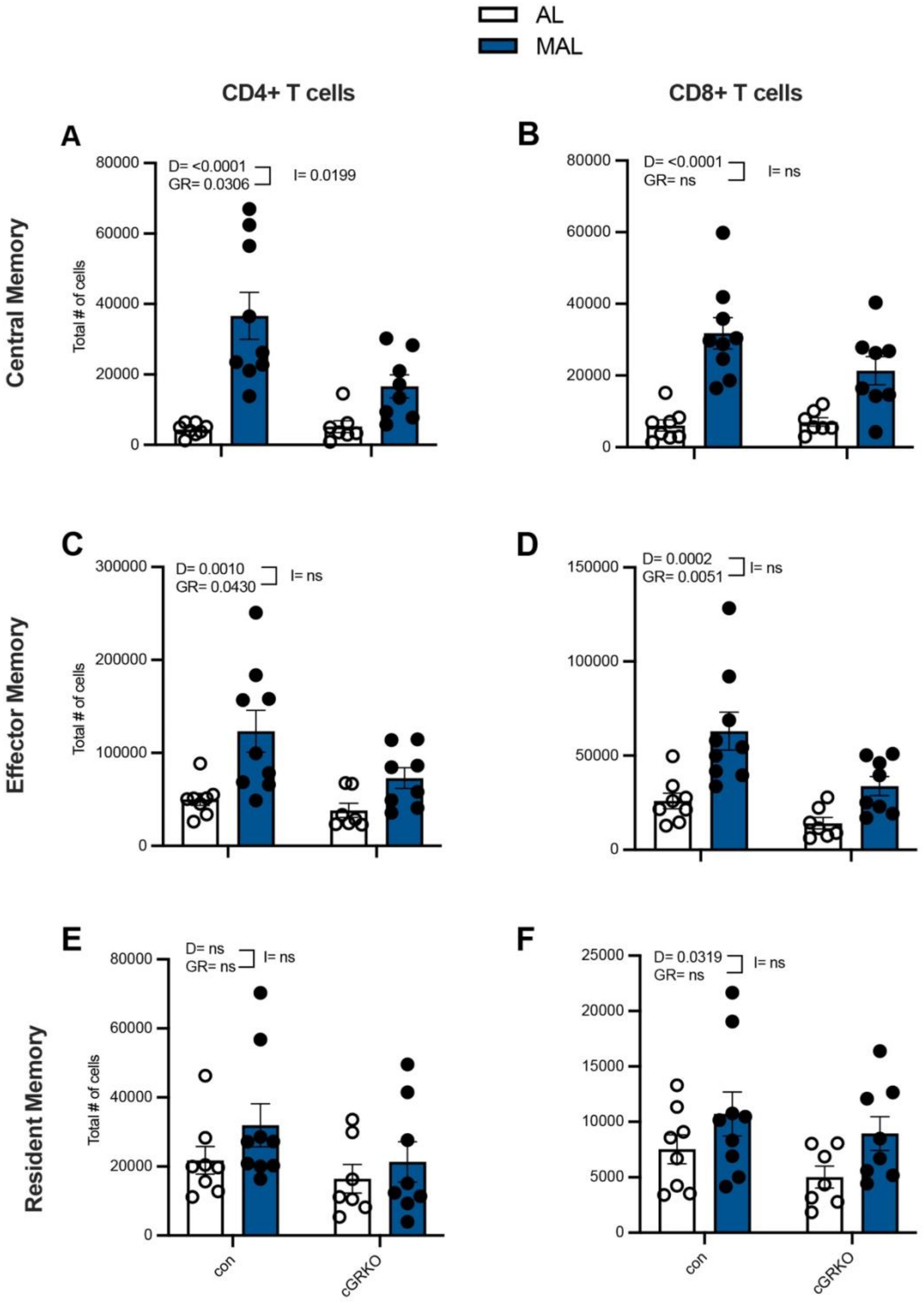

Fig. S3. T cell expression of glucocorticoid receptor (GR) is required for malnutrition-induced increases in effector memory T cells in the bone marrow. Cells were isolated from the bone marrow of control (AL) and malnourished (MAL) mice conditionally lacking the glucocorticoid receptor in T cells (cGRKO) or controls (con) and the percentage

Biology Open • Supplementary information

of central memory (CD44^{hi} CD69- CD62L^{hi}), effector memory (CD44^{hi} CD69- CD62L^{lo}), and resident memory (CD44^{hi} CD69+ CD62L^{lo}) T cells was identified using flow cytometry. Percentages were multiplied by total numbers to yield the number of central memory CD4+ T cells (A), central memory CD8+ T cells (B), effector memory CD4+ T cells (C), effector memory CD8+ T cells (D), resident memory CD4+ T cells (E), and resident memory CD8+ T cells (F). *P* values were obtained by two-way ANOVAs and are displayed on graphs, with GR indicating the effect of glucocorticoid receptor deficiency, D indicating the effect of diet, and I indicating an interaction between both variables. n.s. - not significant. Control: MAL *n*=9, AL *n*=8. GRKO: MAL *n*=8, AL *n*=7. Graphs display means ± s.e.m.

Biology Open • Supplementary information

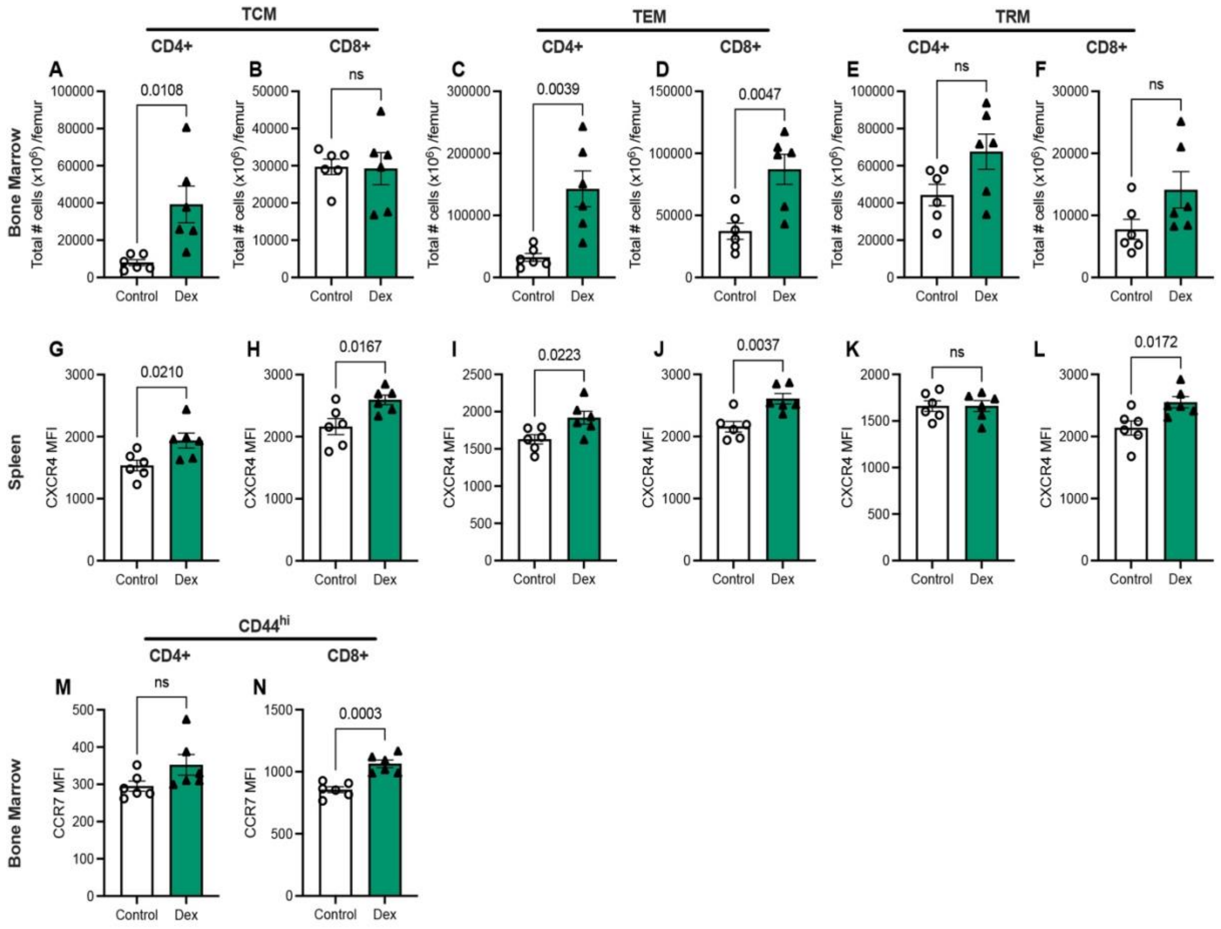

Fig. S4. Glucocorticoids are sufficient to increase the number of effector memory T cells in the bone marrow. Cells were isolated from the bone marrow of mice injected with dexamethasone (2 mg kg^{-1}, resuspended in DMSO and diluted with sterile saline) or a similar DMSO/saline mixture (control) and the percentage of central memory (CD44^{hi} CD69- CD62L^{hi}), effector memory (CD44^{hi} CD69- CD62L^{lo}), and resident memory (CD44^{hi} CD69+ CD62L^{lo}) T cells was identified using flow cytometry. Percentages were multiplied by total numbers to yield the number of central memory CD4+ T cells (A), central memory CD8+ T cells (B), effector memory CD4+ T cells (C), effector memory CD8+ T cells (D), resident memory CD4+ T cells (E), and resident memory CD8+ T cells (F). Flow cytometry was also used to assess expression of CXCR4 on spleen cells (G-L) and CCR7 on bone marrow CD44hi cells (M-N). Individual values are represented by black triangles (dexamethasone) or open circles (control). *P* values were obtained by two-tailed unpaired *t*-tests and are displayed above the corresponding graph. n.s. - not significant. Graphs display means ± s.e.m. n=6/group.

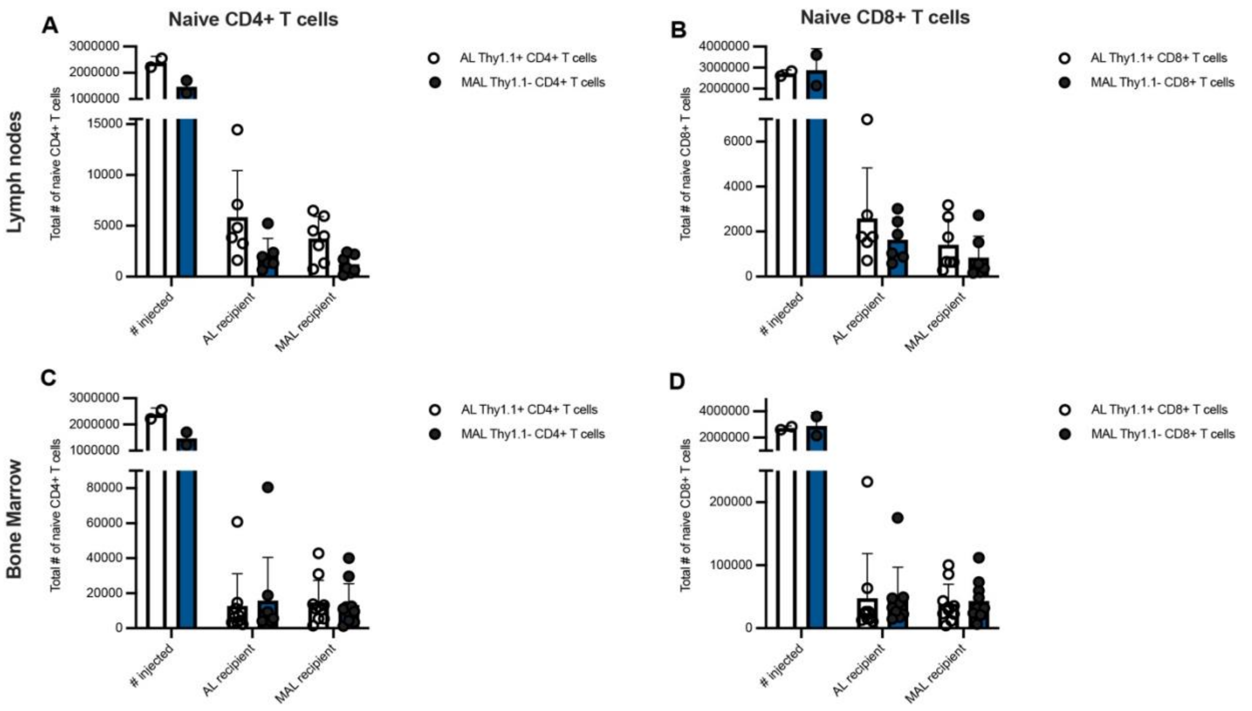

Fig. S5. Total number of cells injected and recovered in adoptive transfer experiments. Pan naive T cells were isolated from (MAL, Thy1.1-) and *ad libitum*-fed (AL, Thy1.1+) donor mice, mixed in a 50:50 ratio, and labelled with CFSE before retro-orbital injection into AL (Thy1.1-) or MAL (Thy1.1-) recipients. Flow cytometry was used to determine the percentage of naïve CD4+ and CD8+ T cells in the mixture before injection (# injected) as well as two hours later on samples recovered from the lymph nodes (A and B) and bone marrow (C and D). The percentages were multiplied by the total number of cells isolated in each tissue to determine the total number of cells. The data from two independent experiments is shown. Lymph node *n*=10/ group. Bone marrow *n*=16/group. Lymph node samples in which the total number of recovered CFSE+ cells was less than 1,500 were excluded from further analysis. Graphs display means ± s.e.m.

Biology Open • Supplementary information
